# Severe Flexor Digitorum Profundus Muscular Adhesion by Pseudo-Volkmann's Contracture without Fracture: A Case Report and Literature Review

**DOI:** 10.1055/a-2398-9052

**Published:** 2024-11-13

**Authors:** Jae Woo Kim, Jong Chan Kim, Sung Hoon Koh, Jin Soo Kim, Si Young Roh, Kyung Jin Lee, Dong Chul Lee

**Affiliations:** 1 Department of Plastic and Reconstructive Surgery, Gwangmyeong Sungae General Hospital, Gwangmyeong, Korea; 2 Department of Plastic and Reconstructive Surgery, National Police Hospital, Seoul, Korea

**Keywords:** pseudo-Volkmann's contracture, flexor tendon, posttraumatic adhesion

## Abstract

Volkmann's ischemic contracture is a condition characterized by permanent ischemic damage to muscles and nerves due to vascular insufficiency, resulting in flexion contractures of the affected limb. In contrast, pseudo-Volkmann's contracture presents with similar clinical features but lacks ischemic damage and has the potential for complete recovery. We report a case of a 39-year-old man who developed failure of extension in the middle and ring fingers of the left hand following blunt forearm trauma from a rolling machine. Despite no skin breakage or fracture, his symptoms progressively worsened over 2 months without treatment. Surgical exploration 2 years later revealed severe adhesions of the flexor digitorum profundus muscle at the myotendinous junction to the ulnar periosteum, with immediate recovery after release. This case highlights pseudo-Volkmann's contracture in an adult without fracture, likely due to blunt trauma causing delayed adhesion formation.

## Introduction

Volkmann's ischemic contracture is a condition caused by vascular insufficiency and ischemia leading to permanent damage to the muscles and nerves. 1 2 3 It commonly arises from acute compartment syndrome induced by fractures but can also be precipitated by other conditions that compromise blood flow, such as tight bandaging, neoplasms, or arterial emboli. 3 4 5 It can affect both the upper and lower limbs. In the upper extremities, it primarily impacts the deep flexor muscle group, including the flexor digitorum profundus (FDP) and flexor pollicis longus, as well as the median nerve. 1 5 6 7 This damage results in muscle shortening and characteristic deformities. Typical findings consist of elbow flexion, forearm pronation, wrist flexion, thumb flexion and adduction, digital metacarpophalangeal joint extension, and interphalangeal joint flexion. The combination of metacarpophalangeal joint extension and proximal interphalangeal joint flexion creates a claw-hand deformity. 2 5 6 8 While deformities may initially be flexible, particularly in milder cases, chronic muscle imbalance and immobility can ultimately lead to a fixed contracture deformity.

In 1976, Jeffery reported a distinct clinical entity resembling Volkmann's ischemic contracture but differing in its pathophysiology. 9 The two pediatric cases described involved both forearm bone fractures due to trauma. After fracture healing, the patients exhibited significant extension limitation from the middle to the little finger, most pronounced in the ring finger. 9 Clinically, no joint deformities were present. While the wrist and metacarpophalangeal joints maintained a straight posture, there was a lack of extension beyond 45 degrees at the proximal interphalangeal joints, though full passive movement was possible with wrist flexion. 9 Surgical exploration revealed the FDP tendon adhered to the ulnar fracture site via a broad fibrous band. 9 Crucially, in contrast to Volkmann's ischemic contracture, no ischemic changes were observed in the muscles or nerves intraoperatively. Surgical release of the adherence between the ulna and FDP tendon resulted in immediate, complete recovery of range of motion. 9 Based on these distinguishing features of entrapment without ischemia and potential for full recovery, in 1979, Baudet and Lafond termed this condition “pseudo-Volkmann's contracture.” 10

We identified an adult patient whose clinical presentation was similar to those described by Jeffery, yet without any accompanying fractures. 9 He was a 39-year-old man who complained of severe extension failure of the middle and ring fingers. Two years prior to presenting at our hospital, the patient had a history of blunt trauma caused by a rolling machine to the left forearm. Due to the absence of skin breakage and fractures, the primary hospital did not consider surgical intervention and instead administered immobilization and physical therapy for 2 weeks. The patient exhibited a gradual worsening of symptoms during the first 2 months following the injury. He lived a normal life without further deterioration for 2 years, after which he sought consultation at our institution. On physical examination, failure of extension was observed in the proximal and distal interphalangeal joints of the middle and ring fingers. Full passive extension was achievable when the wrist was flexed, indicating that the joints were supple. No sensory changes were detected in the median nerve territory. Plain radiographs revealed no evidence of a fracture in the forearm.

## Case

A 39-year-old male industrial worker presented to the clinic with complaints of difficulty extending the middle and ring fingers. The patient stated a history of blunt compression injury to the left mid-forearm 2 years prior while operating a rolling machine.

According to the medical record from the primary hospital, the patient exhibited painful swelling and bruising in the forearm without apparent open wounds. During the initial physical examination, muscle function was found to be normal but pain, and the patient did not report any sensory changes. The initial treatment included immobilization for 1 week, management of pain, and conservative measures for the bruising and swelling. Surgical intervention was not considered at the time of injury because of the absence of open wounds or bone fractures. Owing to personal circumstances, the patient discontinued treatment and returned to the workplace following relief from pain. A gradual worsening in the limitation of digital extension occurred without painful events over the next 2 months, though no explicit documentation of hand motions was provided. The patient endured 2 years with contractures affecting both the middle and ring fingers without deterioration, during which no further treatment was pursued.

**Fig. 1:**
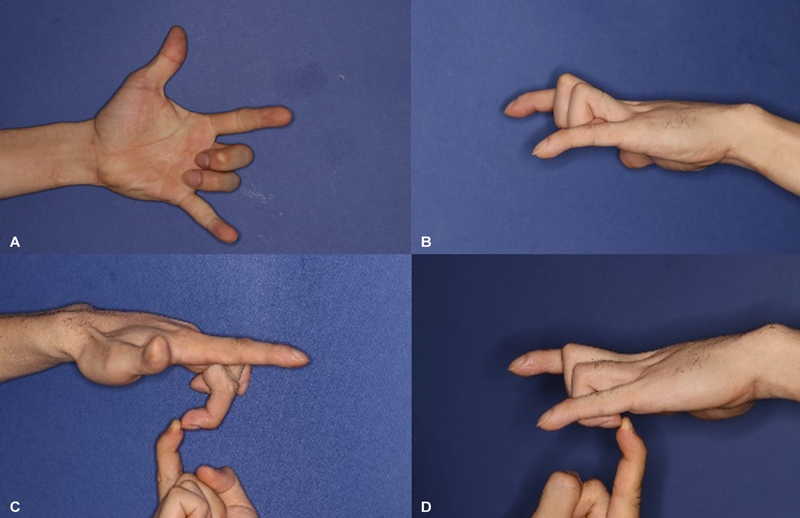
Preoperative contracture in the ring and middle fingers. ( **A, B** ) Preoperative active range of motion (ROM). ( **C, D** ) Preoperative passive ROM.

He visited our clinic, reporting difficulty in extending the middle and ring fingers, without any accompanying pain or neurological symptoms. Physical examination revealed failure of extension of the distal and proximal interphalangeal joints in both the middle and ring fingers ( Fig. 1 ), while full flexion was preserved. Full extension of the involved digits was achieved in response to wrist flexion, demonstrating the suppleness of the digital joints. No scars were evident from open wounds. Upon palpation of the injured area, mobilization of the muscle distally was found to be restricted. A mild decrease in power grip strength was recorded at 65 lb, in comparison to 85 lb on the unaffected side, while the sensory evaluation remained normal. Plain radiographs demonstrated no evidence of a fracture or periosteal reaction, except for an old fracture at the base of the third metacarpal. No further imaging studies were performed. Informed consent was obtained from the patient prior to all procedures and research.

### Surgical Treatment

Based on the presumptive diagnosis of adhesion of the deep flexor muscle group to the forearm, surgical exploration was planned to precisely locate the lesion. Owing to the challenge of identifying the precise location of the adhesion, a lazy-Z incision extending 15 cm proximal and distal to the site of injury was designed. There were no signs of injury from the skin to the subcutaneous layer. No remarkable findings were noted within the muscle fascia, and no major adhesions were observed in the flexor digitorum superficialis (FDS) muscle belly or tendon. The median nerve was found to be unaffected. Between the FDS muscle and the FDP muscle belly and tendon, no adhesions were seen and the FDS movement was not restricted. However, the FDP muscle belly and tendon showed limited motion.

**Fig. 2:**
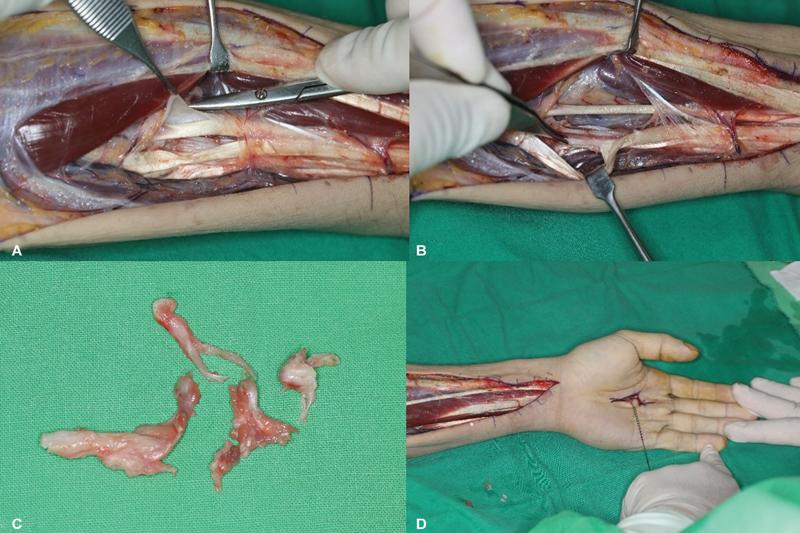
( **A** ) Severe adhesions of the flexor digitorum profundus tendon group. ( **B** ) Innocent median nerve. ( **C** ) Resected hypertrophic fascia. ( **D** ) Immediate improvement of extension in the middle and ring fingers.

The exposed FDP of the index finger was found to be unremarkable, and access was made to the radial side of the deep flexor muscle belly and tendon. The area between the bone and the interosseous septum was inspected at the level of the myotendinous junction. Tightly adhered whitish scar tissue was encountered on the fascia at the site where the FDP myotendinous junction of the middle and ring fingers met the ulnar bone surface ( Fig. 2A ). The median nerve was found to be unremarkable ( Fig. 2B ). For the FDP of the little finger, gliding and a full range of motion were confirmed. The extent of the adhesion was identified as 7 cm long, and it was completely released through surgical excision ( Fig. 2C ). Subsequently, immediate improvement in the motion of the middle and ring fingers was achieved ( Fig. 2D ). Additional dissection was performed at the palmar crease to discern tendon adhesions in the palmar area, and no abnormalities were observed in the flexor tendon or surrounding structures. To prevent hematoma formation, a drain was placed, and the surgical site was closed.

### Postoperative Course

**Fig. 3:**
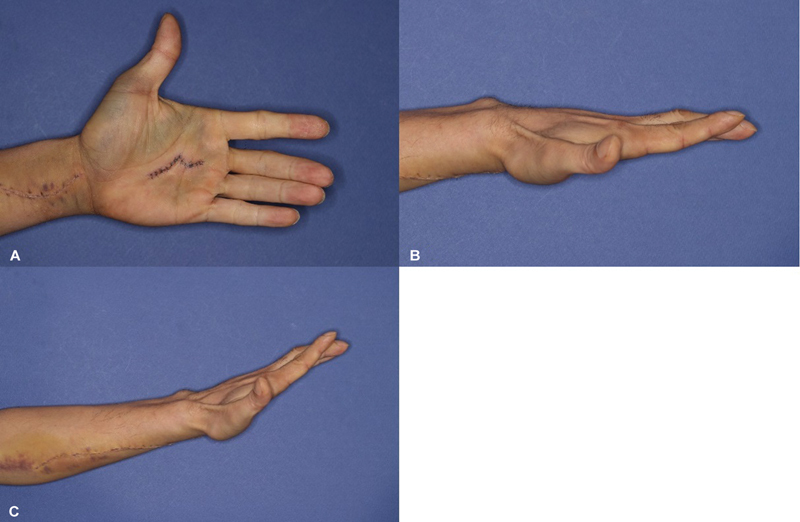
( **A, B** ) Range of motion in the middle and ring fingers during follow-up at postoperative day 7 showing the ability to extend the involved fingers. ( **C** ) Although the wrist is extended, slight limitations were observed due to pain. However, passive extension was unimpeded in the affected digits.

Postoperatively, a splint was not applied, and the patient was instructed and encouraged to perform self-oriented active motion. A full range of motion was maintained throughout the 3-week hospitalization ( Fig. 3 ). Furthermore, no signs of muscular contracture were observed. There were no wound complications, and the patient was discharged uneventfully, returning to the workplace.

### Postoperative Complication and Management

**Fig. 4:**
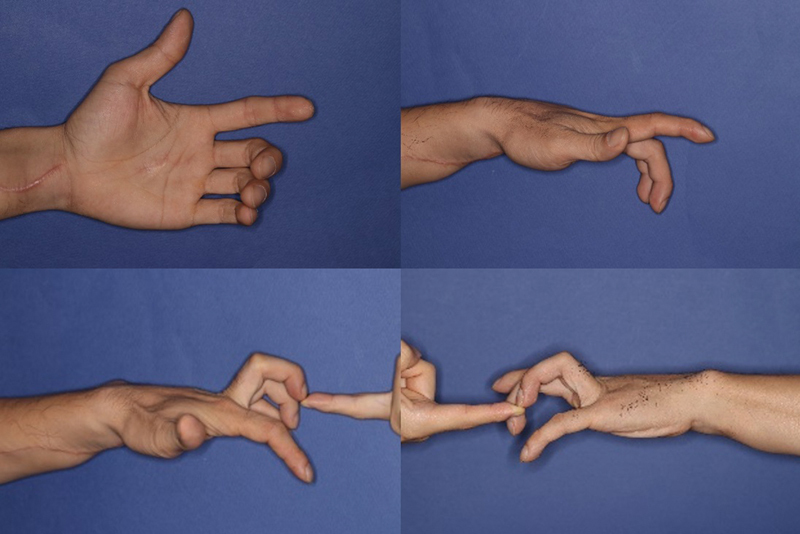
Contracture recurs mostly in the middle and ring fingers, which was associated with pain in the forearm and transient sensory deficit on the affected hand 2 months after the primary operation.

Two months postoperatively, the patient experienced worsening pain in the mid-forearm, linked to a history of forceful pulling at work. He reported difficulty extending all fingers, with intermittent sensory changes in the median nerve territory, including numbness and tingling ( Fig. 4 ). Physical examination revealed soft tissue swelling and a firm, palpable mass in the mid-forearm, causing severe pain upon palpation.

**Fig. 5:**
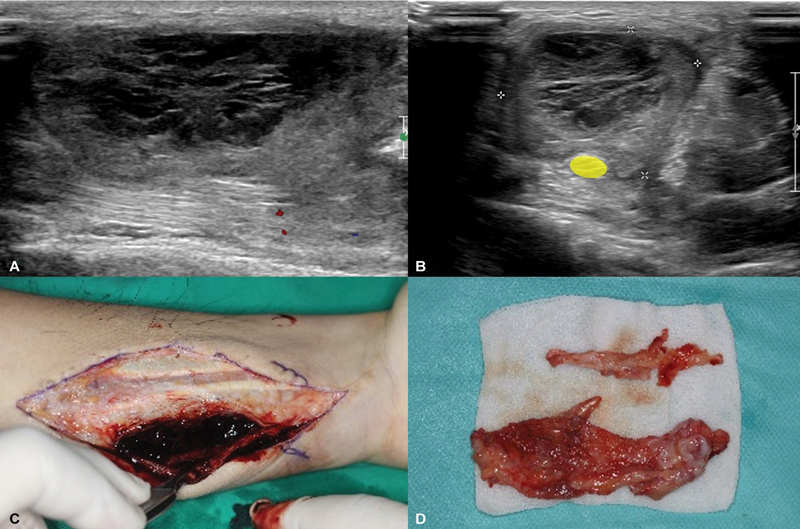
( **A** ) Ultrasonography reveals hematoma at the proximal one-third of the left flexor digitorum superficialis (FDS) muscle. ( **B** ) Compression of the median nerve (yellow mark) by encapsulated hematoma. ( **C** ) Hematoma adhered to both FDP and FDS muscle groups. ( **D** ) Resected FDS fascia. FDP, flexor digitorum profundus.

Radiography showed soft tissue swelling and an indistinct, oval-shaped area at the distal one-third level. Ultrasonography identified a 5.5-cm encapsulated hematoma compressing the median nerve at the proximal forearm level ( Fig. 5A, B ). The patient was taken to the operating room for reexploration.

During surgery, no active bleeding was found. The hematoma was encapsulated between the FDS and FDP tendon groups. Significant adhesions and fascial hypertrophy were observed, restricting movement in the FDP and FDS tendons ( Fig. 5C ). The encapsulated hematoma and organized capsule were removed, alleviating compression on the median nerve ( Fig. 5D ). The source vessel was not precisely identified but small vessels along the fascia were cauterized.

**Fig. 6:**
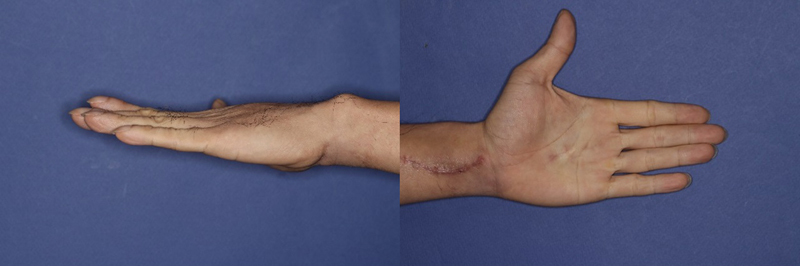
Improvement of extension in the involved fingers after 3 weeks.

Postoperatively, pain and numbness resolved, and two-point discrimination was 5 mm in both static and moving tests, indicating normal sensory function. Grip strength measurements showed diminished power on the affected side but self-directed exercises restored full finger flexion and extension. After 3 months of targeted therapy, the strength of the involved fingers returned to normal ( Fig. 6 ).

## Discussion

The distinguishing features of pseudo-Volkmann's contracture, in contrast to Volkmann's ischemic contracture, can be summarized into two major aspects. Firstly, the restriction of movement in the muscles and tendons is localized, resulting mainly from the entrapment of muscles and tendons by bone spikes due to fractures and the formation of scar tissue leading to adhesions during the healing process at the injury site. 6 9 11 12 13 Secondly, there is no permanent ischemic damage to muscles and nerves, with function being preserved. 9 10 13 This aspect allows for the anticipation of immediate recovery of the range of motion following surgical release.

Twenty-eight cases have been documented in the English medical literature since pseudo-Volkmann's contracture was first reported in 1976. 6 9 10 11 12 13 14 Predominantly, these cases involved post-radioulnar fractures in pediatric patients aged less than 19 years. 13 The typical clinical presentation often manifests as a failure of extension in the distal and proximal interphalangeal joints. The affected fingers can range from one to four, excluding the thumb, with involvement of the ring finger being most commonly observed. 13 This is due to the anatomical correlation where the fixed structures, the ulna, and the interosseous membrane, are in proximity to the tendons of the FDP for the middle and ring fingers. 9 10 For the same reason, cases involving the FDS tendon are almost nonexistent. 13 When the wrist is in a straight position, both active and passive extension are limited; however, when the wrist is flexed, reducing tension on the flexor tendons, full extension of the affected fingers is observed. 6 9 This indicates that the joints remain supple, and no abnormalities are noted in the intrinsic function of the hand. Depending on severity, the duration from injury to treatment ranged between 2 days and 16 years. Bone spikes from fractures or adhesions formed during healing may have caused limitation of movement around the myotendinous junction of the deep flexor group. 13

Our case differed from previous reports in that there was no concomitant fracture of the radius or ulna, and the symptoms worsened over a relatively extended period of 2 months posttrauma. The development of contractures without fractures could be attributed to the characteristics of the injury mechanism. The patient experienced a short and forceful compression from a rolling machine, which traversed the skin and soft tissues without causing any damage until it encountered the rigid ulnar bone. Here, the energy was concentrated, resulting in bleeding from the small vessels of the periosteum, localized to the area surrounding the ulnar surface, while leaving the skin and other soft tissues intact. This might have initiated adhesions at the myotendinous junction of the FDS, directly in contact with the surface of the ulna, gradually exacerbating the symptoms over a 2-month period. When the patient visited our hospital, a CT scan was not performed. However, conducting a CT scan before surgery could have been beneficial in identifying the presence, extent, and severity of tendon adhesions, thus aiding in surgical planning.

The prognosis for full recovery is excellent when pseudo-Volkmann's contracture is promptly identified and treated. Treatment should include re-manipulation with extension of the fingers or open surgical release of the adhered muscle belly. 9 11 13 15 For prevention, it is crucial to perform a thorough examination during the initial visit or at the end of immobilization in cases with fractures. Prompt intervention should be undertaken if any suspicion arises. If stiffness or pain occurs, intensive physiotherapy with finger extension exercises is recommended to prevent extension lag caused by chronic adhesion formation. The extension exercises should be conducted weekly for 6 weeks, accompanied by a passive stretch test of the ring finger every week during this period. During these exercises, the wrist and metacarpophalangeal joint should be at maximum extension, and the ring finger should be passively extended to hyperextension at full wrist extension. 14 In case of a lack of careful examination, diagnosis of flexor tendon entrapment may be delayed until cast removal or even years later. Missed opportunities for nonsurgical improvement can be particularly detrimental in adult patients, as adhesion of the myotendinous unit and contracture of the proximal interphalangeal joints tend to develop more rapidly with delayed diagnosis. 11 13 14 15 Although this condition has resolved spontaneously in a few patients, prompt surgical treatment can prevent complications by detecting those at imminent risk.

Treatment of pseudo-Volkmann's contracture is challenging with the application of a single method. The treatment approach at the primary hospital in this case, limited to pain control and warm therapy, proved insufficient in preventing the progressive development of contracture, which worsened over 8 weeks posttrauma. Therefore, close follow-up of all forearm injuries, especially those involving the proximal third, is imperative, even in cases where immediate posttraumatic symptoms such as limitation of motion, fractures, or open wounds are not present. Treatment should consist of weekly passive stretching tests and instruction on dynamic finger extension exercises. 13 14
